# Primary Cutaneous Aspergillosis Due to Aspergillus flavus After Nasal Packing in a Neutropenic Patient With Acute Myeloid Leukemia: A Case Report

**DOI:** 10.7759/cureus.113066

**Published:** 2026-07-21

**Authors:** Nidae Mimouni, Jinane Bari, Siham Karrati, Imane El Khannouri, Awatif El Hakkouni

**Affiliations:** 1 Parasitology-Mycology Laboratory, Mohammed VI University Hospital, Marrakech, MAR; 2 Research Laboratory of Infectious Disease Control, Faculty of Medicine and Pharmacy, Cadi Ayyad University, Marrakech, MAR

**Keywords:** acute myeloid leukemia, aspergillus flavus, invasive fungal infection, neutropenia, primary cutaneous aspergillosis

## Abstract

Primary cutaneous aspergillosis (PCA) is an uncommon mycosis resulting from direct inoculation of *Aspergillus* into the skin. We report the case of a 33-year-old woman with acute myeloid leukemia (AML) who developed a rapidly progressive paranasal erythematous lesion after induction chemotherapy, febrile neutropenia, and anterior nasal packing for epistaxis. Despite broad-spectrum antibiotics, the lesion evolved into a violaceous necrotic plaque with facial cellulitis and nasal obstruction. Direct microscopy showed hyaline septate hyphae with acute-angle branching and culture identified *Aspergillus flavus*. Histopathology confirmed tissue invasion by fungal elements. The patient improved after voriconazole therapy following neutrophil recovery, with complete clinical healing. This case shows that early mycological diagnosis and rapid antifungal therapy are essential in immunocompromised patients with necrotic skin lesions.

## Introduction

Aspergillosis is an opportunistic mycosis caused by molds in the genus *Aspergillus*, predominantly *Aspergillus fumigatus *(*A. fumigatus*)* *and *Aspergillus flavus *(*A. flavus*) [1]. Risk factors for aspergillosis include granulocytopenia, diabetes mellitus, local tissue trauma, the newborn period, hematologic malignancies, and congenital or acquired immunosuppression [2].

Cutaneous aspergillosis may be primary, when the skin is the initial site of infection, or secondary, when skin involvement results from hematogenous dissemination of the disease after an initial pulmonary infection [1]. Distinguishing primary from secondary cutaneous aspergillosis relies on clinical context, histology, and the exclusion of a deeper or disseminated focus, and this distinction affects management and prognosis [1,3].

Although the incidence of primary cutaneous aspergillosis (PCA) in immunocompromised patients is not well defined, reported cases appear to be increasing, likely because of improved recognition and the growing population of patients with impaired immunity [3].

Nasal packing may act as a portal of fungal inoculation, since it combines mucosal trauma, occlusion, and a moist environment that promotes fungal germination. In this report, we describe a case of PCA due to *A. flavus* that developed after anterior nasal packing in a patient who had received induction chemotherapy for acute myeloid leukemia (AML). This case illustrates a rare portal of inoculation and the importance of early diagnosis in neutropenic patients.

## Case presentation

A 33-year-old woman was diagnosed with AML. Induction chemotherapy was initiated with daunorubicin and cytosine-arabinoside. During the induction phase, she developed severe epistaxis while in a state of profound febrile neutropenia, which required anterior nasal packing. At that time, the absolute neutrophil count was 4/mm³ with a nadir of 0/mm³ and a platelet count of 16,000/mm³. Four days after removal of the nasal packing, she developed an erythematous, edematous paranasal lesion. The patient was febrile at the onset of the lesion.

**Figure 1:**
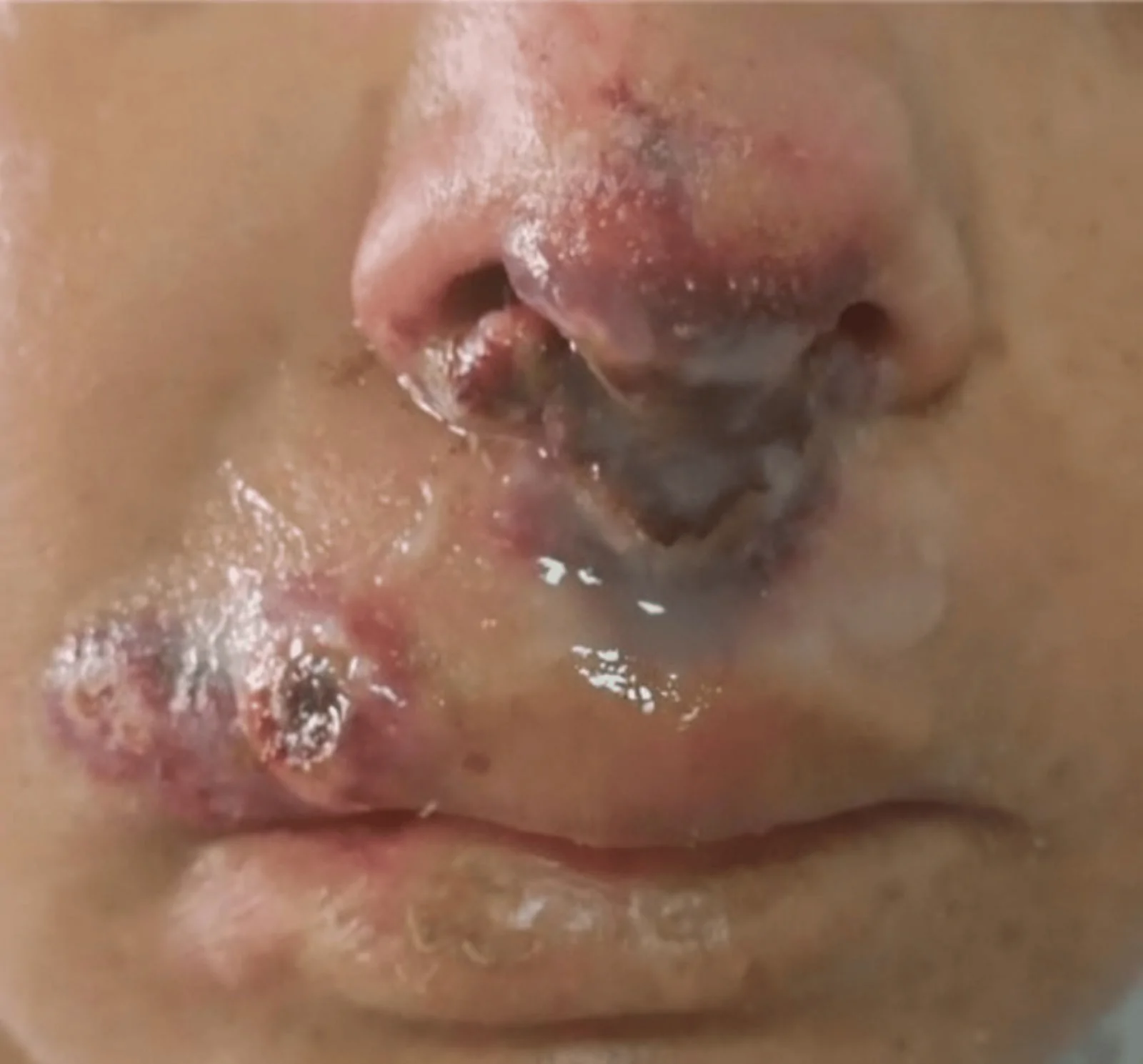
Necrotic paranasal plaque on the right side extending toward the cheek

Despite broad-spectrum antibiotic therapy with piperacillin-tazobactam and vancomycin, the lesion showed no improvement and rapidly evolved into a violaceous necrotic plaque extending toward the cheek (Figure 1). The patient subsequently developed progressive facial cellulitis and nasal obstruction. Computed tomography scan of the sinuses showed complete opacification of the maxillary and ethmoid sinuses raising concern for invasive fungal rhinosinusitis.

**Figure 2:**
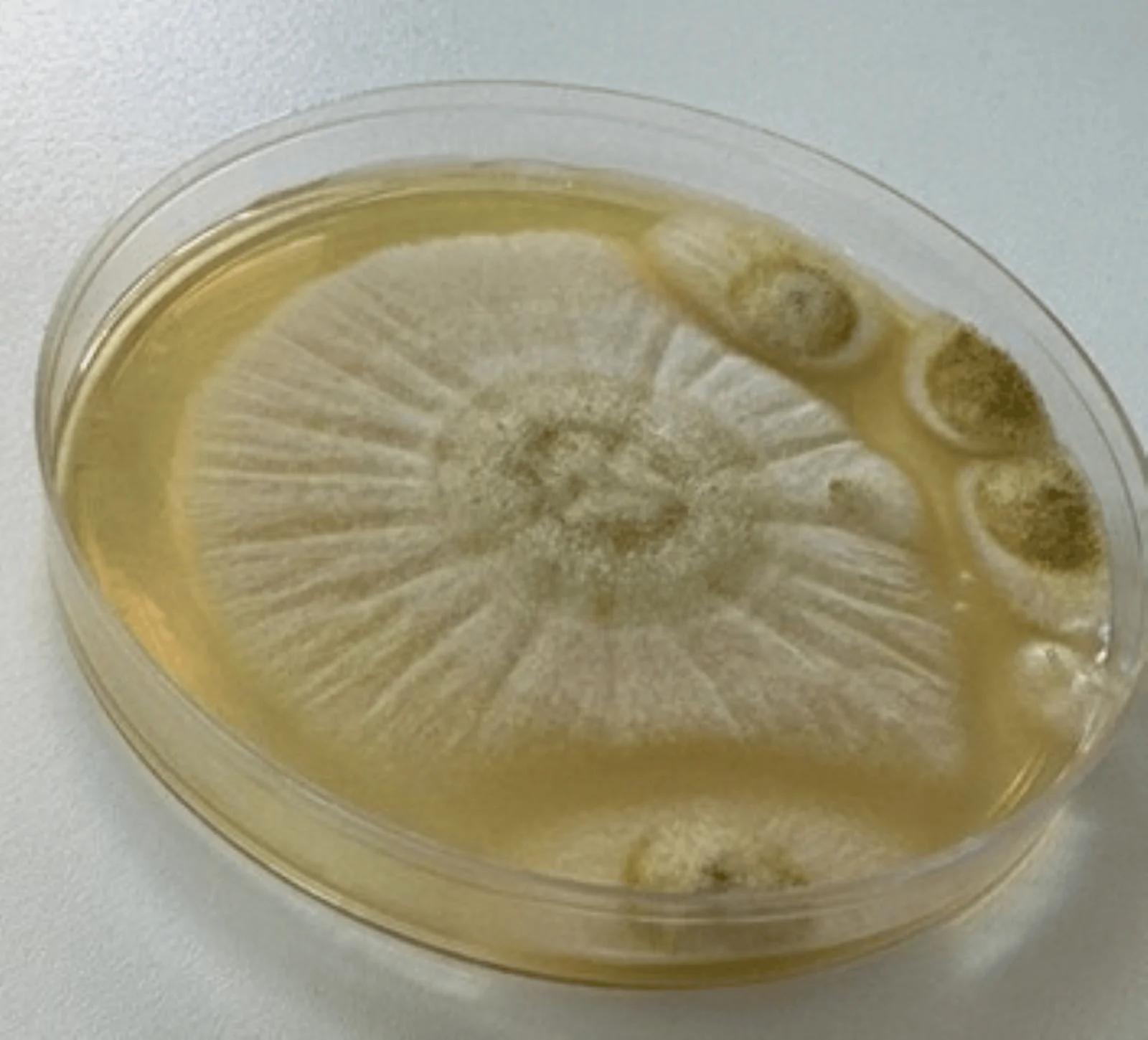
Velvety yellow-green colonies with a white peripheral margin

Direct microscopic examination of the lesion samples was performed with a 10% potassium hydroxide (KOH) mount, May-Grünwald-Giemsa (MGG) staining, and lactophenol cotton blue, which demonstrated hyaline septate hyphae with regular dichotomous branching at approximately 45°, highly suggestive of *Aspergillus* species. Specimens were inoculated onto Sabouraud dextrose agar (SDA) with chloramphenicol and incubated at both 25 °C and 37 °C. Within 48 hours, the cultures yielded rapid growth of velvety, yellow-green colonies with a white peripheral margin (Figure 2).

**Figure 3:**
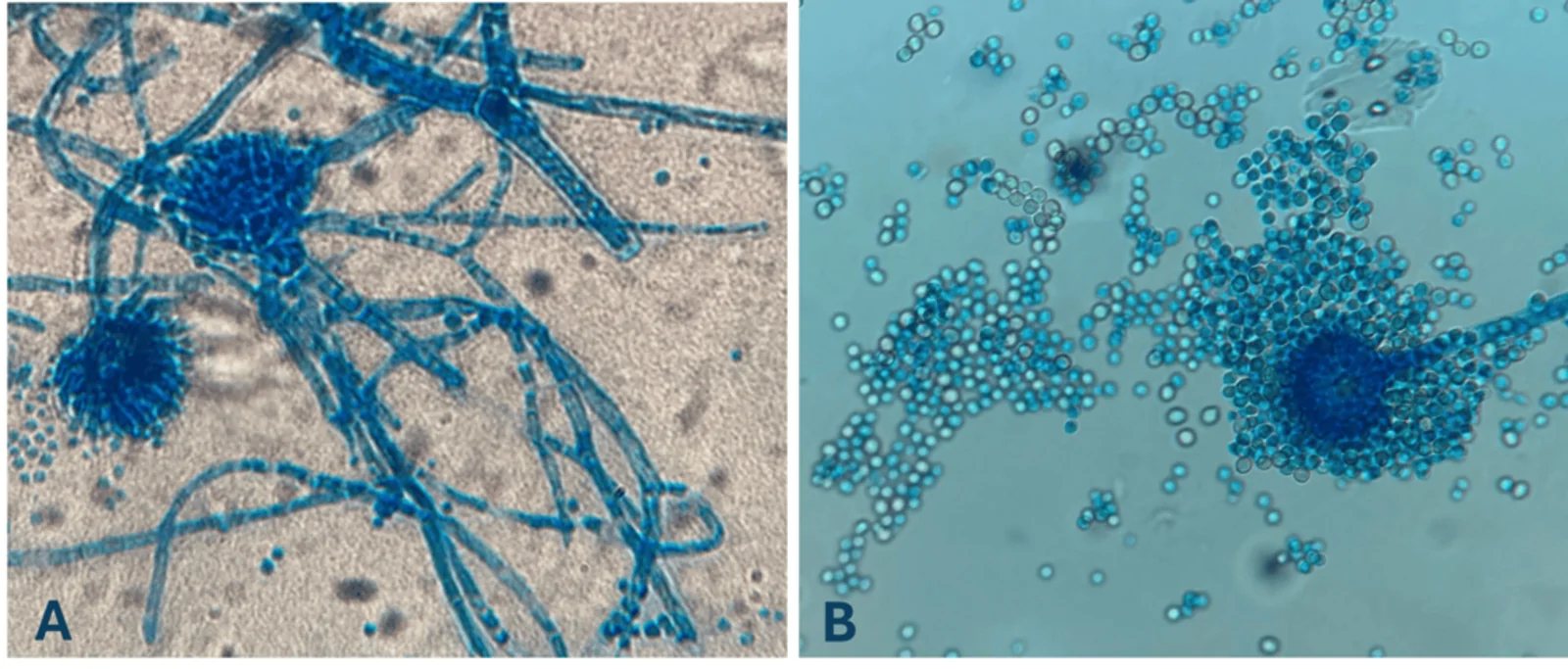
Lactophenol cotton blue preparation showing the morphology of Aspergillus flavus (x400) (A) Conidiophores with biseriate phialides covering the entire vesicle, typical of Aspergillus species. (B) Abundant globose to subglobose echinulate conidia.

Microscopic identification of the isolate confirmed *A. flavus,* characterized by typical conidiophores and radiating conidial heads (Figure 3). 

**Figure 4:**
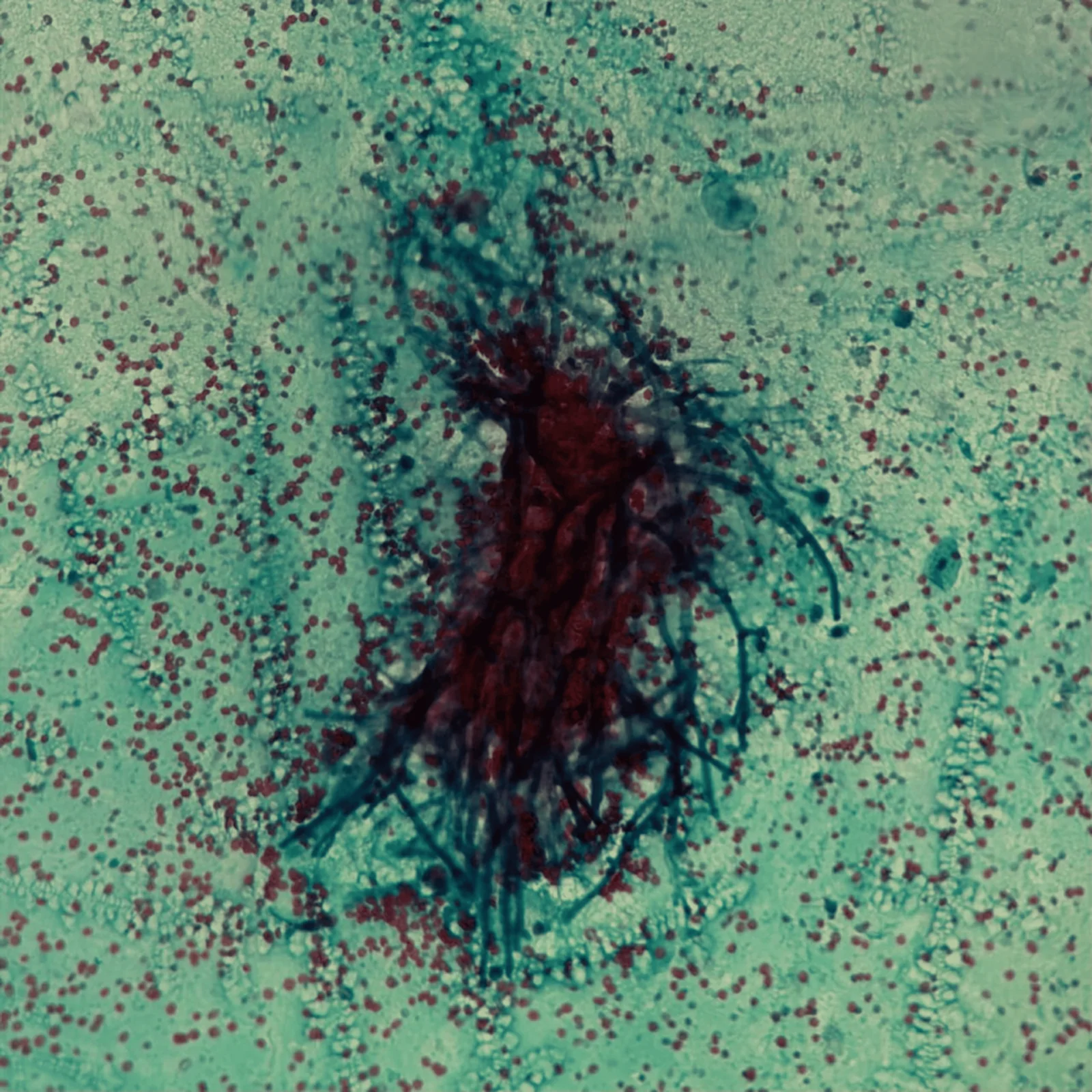
Grocott-Gomori methenamine silver (GMS) stain of the skin biopsy showing a mass of branching fungal hyphae consistent with Aspergillus species (high-power field)

Histopathological analysis of a skin biopsy further confirmed the presence of invasive fungal elements within the dermis, consistent with the mycological findings (Figure 4).

**Figure 5:**
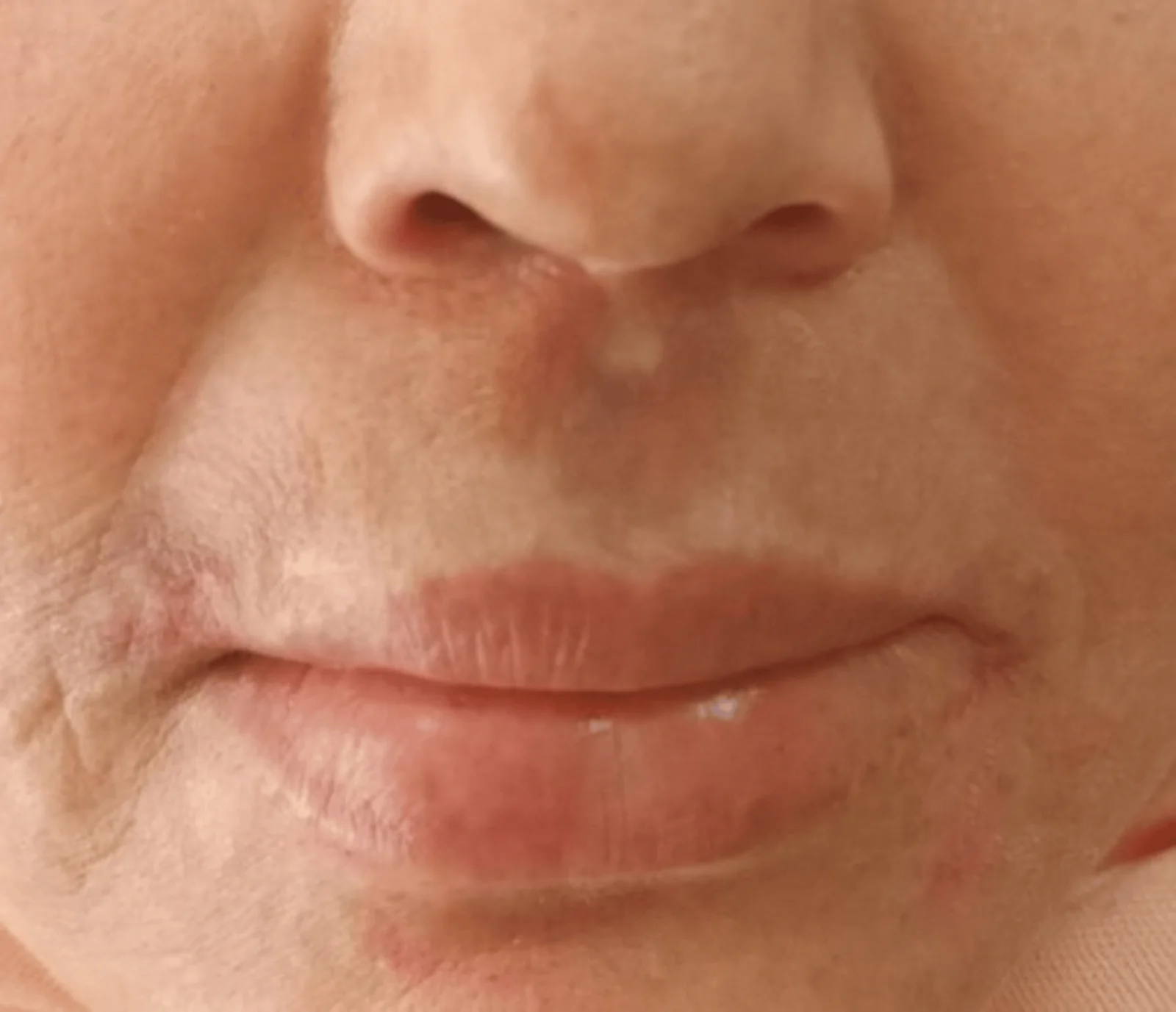
Clinical improvement with lesion resolution after therapy

The patient was treated with voriconazole at standard doses (loading dose of 6 mg/kg every 12 h, followed by 4 mg/kg every 12 h) for approximately 10 weeks. After initiation of antifungal therapy and subsequent neutrophil recovery, the facial lesions showed significant regression and complete clinical healing (Figure 5). Antifungal therapy was discontinued after neutrophil recovery, a decrease in C-reactive protein, and complete healing of the cutaneous lesion.

## Discussion

In patients with hematological malignancies, fungal infections are a major source of morbidity and mortality. In this population, aspergillosis ranks as the second most common life-threatening mycosis [4]. It is associated with elevated hospital mortality, extended duration of hospitalization, and high costs [5].

*Aspergillus* is a large genus of ubiquitous saprophytic molds found in soil, decaying vegetation, and air. These fungi can cause various superficial infections, including otomycosis, distal lateral and proximal subungual onychomycosis, and cutaneous aspergillosis [6].

Invasive aspergillosis occurs in a context of compromised immune defenses, impacted by environmental or iatrogenic factors and enhanced by the pathogen’s capacity to bypass the immune system. *Aspergillus* forms biofilms that shield it from both phagocytosis and antifungal drugs. By modifying its cell wall components, the fungus avoids recognition by pattern recognition receptors (PRRs), reducing the host's ability to detect and eliminate fungal cells [7]. 

Profound neutropenia, commonly found in most patients with hematological malignancies, remains one of the principal risk factors for this disease by depriving the immune system of the phagocytic response required to inhibit fungal invasion [6,8]. Additional immunosuppressive treatments increase vulnerability [4,9,10]. Posaconazole prophylaxis is recommended in high-risk patients [11].

The clinical forms of aspergillosis depend on the infecting *Aspergillus* species, the site of infection, and the host's immune status. This variability reflects the different interactions between the fungus and the host immune system [7]. Invasive aspergillosis, the most aggressive form, mainly involves the lungs. Chronic pulmonary aspergillosis (CPA) is a less aggressive form of disease that affects immunocompetent patients with pre-existing lung conditions such as chronic obstructive pulmonary disease (COPD), tuberculosis, or sarcoidosis. Allergic bronchopulmonary aspergillosis (ABPA) occurs mainly in patients with asthma or cystic fibrosis [7]. *Aspergillus* can disseminate beyond the lungs, causing sinusitis, keratitis, and endocarditis [7].

Cutaneous aspergillosis is an infrequent but severe, locally invasive mycosis that may present as either a primary or a secondary infection. PCA usually develops where the fungus has been directly inoculated into the skin [3]. It requires the combination of skin barrier disruption and immunosuppression. In neutropenic patients, *Aspergillus* spores deposited on injured skin germinate into hyphae that invade blood vessels causing ischemia, thrombosis, and necrotic skin lesions. Secondary forms result either from hematogenous dissemination or direct extension from nearby infections, particularly from the lungs or paranasal sinuses [3,12]. 

Localized tissue damage constitutes a portal of entry for opportunistic fungi and provides a site of spore germination. Medical interventions or traumatic breaches disrupting the skin or mucosal barrier enable direct inoculation, while localized occlusive conditions promote angioinvasion and tissue necrosis [1,7,13].

Colony morphology depends on the culture medium, incubation conditions, and *Aspergillus* species. *A. flavus *is commonly cultured on standard fungal media such as Sabouraud dextrose agar (SDA). On this culture medium, it grows rapidly, forming velvety to granular colonies that evolve from pale yellow to yellow-green or olive-green, with a pale to cream-colored reverse.

Direct microscopic examination represents a fundamental step in the diagnosis of *Aspergillu*s infections. Typically, it reveals septate hyaline hyphae with regular contours, showing dichotomous branching at acute angles of approximately 45°. *A. flavus* is characterized by globose to subglobose vesicles bearing radiating phialides that cover the entire surface. The species is mainly biseriate, although uniseriate forms may occur [9].

The identification of the *Aspergillus* genus and its primary species (*A. fumigatus, A. flavus*) can be more specific using polymerase chain reaction (PCR) techniques targeting regions such as the internal transcribed spacer (ITS) or through matrix-assisted laser desorption ionization-time of flight (MALDI-TOF) mass spectrometry. Furthermore, the detection of circulating antigens, such as galactomannan or (1-3)-β-D-glucan, allows for an early diagnosis in immunocompromised patients [14-16].

Published cases of cutaneous aspergillosis show recurrent epidemiological, clinical, and microbiological patterns that support our observation. *A. flavus* has been reported as the main pathogen in several studies [2,12]. Clinically, lesions progress from erythematous macules or papules to necrosis, hemorrhagic bullae, and eschars reflecting the angioinvasion [1,6,9]. Failure of antibacterial therapy with worsening necrosis is a warning sign requiring biopsy and fungal cultures [1,3].

Current guidelines recommend voriconazole or isavuconazole as first-line therapy for invasive aspergillosis, replacing amphotericin B deoxycholate after randomized trials showing better outcomes and improved survival with voriconazole [11]. In line with this guideline, our patient was treated with voriconazole. Previous reports confirm its effectiveness in neutropenic patients [3,12]. Surgical debridement plays a complementary role by reducing fungal burden and improving antifungal penetration. Treatment duration should be individualized and guided by immune recovery, particularly neutrophil restoration [1,3,17].

In this context, localized disease refers to *Aspergillus* infection confined to the skin with no other documented site of infection at presentation, while disseminated disease refers to cutaneous lesions accompanying or secondary to infection at other sites and/or fungemia [4].

Prognosis is largely determined by immune status, particularly neutrophil recovery, which is associated with good outcomes in localized disease but high mortality in disseminated forms despite treatment [4]. This was observed in our patient, in whom control of the infection followed neutrophil recovery. In contrast, isolated cases in immunocompetent patients show that trauma-associated infection may remain localized and respond favorably to antifungal therapy [18,19].

Our report has some limitations. Nasal endoscopy and sinus tissue sampling were not performed, and a contiguous sinonasal focus could therefore not be histologically excluded. The sinus opacification on CT may reflect retained secretions after nasal packing. We considered primary cutaneous aspergillosis the most probable diagnosis, based on the direct portal of entry at the packing site, the isolation of *A. flavus* from the cutaneous lesion, the histological demonstration of fungal invasion on the cutaneous biopsy, and the absence of sinonasal symptoms before the skin lesion appeared.

## Conclusions

Cutaneous aspergillosis remains a rare but life-threatening complication in immunocompromised patients. The prognosis depends primarily on early diagnosis and effective voriconazole-based therapy. Surgical debridement of necrotic tissue is reserved for selected or refractory cases. In the present patient, the cutaneous lesion healed with medical treatment alone. Beyond antifungal therapy, the restoration of immune system remains the most powerful determinant of survival, highlighting the importance of a multidisciplinary approach in this population of patients. This case illustrates the central contribution of the mycology laboratory to both diagnosis and management of cutaneous aspergillosis, from identifying the causative fungus to guiding antifungal treatment.
